# Coronectomy of impacted mandibular third molars: a clinical and radiological retrospective case series study with 2-9 years of follow-up

**DOI:** 10.4317/medoral.26159

**Published:** 2023-08-25

**Authors:** Juan Carlos Bernabeu-Mira, David Peñarrocha-Oltra, Miguel Peñarrocha-Diago

**Affiliations:** 1 Predoctorate Researcher. Oral Surgery Unit, Department of Stomatology. Faculty of Medicine and Dentistry. University of Valencia, Valencia, Spain; 2 Full Professor and Chairman of Oral Surgery. Oral Surgery Unit, Department of Stomatology. Faculty of Medicine and Dentistry. University of Valencia, Valencia, Spain; 3 Professor. Oral Surgery Unit, Department of Stomatology. Faculty of Medicine and Dentistry. University of Valencia, Valencia, Spain

## Abstract

**Background:**

Impacted mandibular third molars occasionally are in intimate relation to the inferior alveolar nerve (IAN). Coronectomy has been proposed as a good alternative to prevent injury of the IAN. The present study evaluates the clinical and radiological outcomes of impacted mandibular third molars presenting radiographic signs associated with a high risk of IAN injury, and which were treated with the coronectomy technique.

**Material and Methods:**

A retrospective case series evaluated the outcomes of coronectomies of impacted mandibular third molars. The inclusion criteria were: available preoperative, immediate postoperative and two-year panoramic radiographs, preoperative cone-beam computed tomography (CBCT), and a complete case history. The clinical evaluation comprised intraoperative complications (mobilized fragments of root and damage to adjacent structures), short-term complications (sensory alterations and postoperative infection), and long-term complications (infection or oral exposure). The IAN position with respect to the roots, root shape, eruption status, third molar position, radicular-complex migration and bone above roots were radiographically evaluated as well.

**Results:**

Approximately a total of 2000 mandibular third molars were removed from 2011 to 2022. Of these, 39 molars in 34 patients were partially extracted using the coronectomy technique. The mean age was 36 years (range 22-77), and the mean follow-up was 28 months (range 24-84). There were two short-term postoperative infections. One of them was resolved through reintervention to remove the roots after antibiotic treatment, while the other required hospital admission and removal of the roots. One case of short-term transient lingual paresthesia was also recorded. Two long-term oral exposures were detected, and the root fragments had to be extracted. There were no permanent sensory alterations.

**Conclusions:**

In our case series of 39 impacted mandibular molars in intimate contact with the IAN and treated with the coronectomy technique, the number of complications was low (two infections and a single case of transient lingual paresthesia), and no permanent sensory alterations were observed. Prospective studies, especially randomized clinical trials, are needed to compare this technique with conventional extraction.

** Key words:**Coronectomy, inferior alveolar nerve injury, mandibular third molar, extraction.

## Introduction

Surgical removal of mandibular third molars is one of the most frequent oral surgeries (1). The mandibular third molar coronectomy technique is still debated among professionals. The roots of the third molar and the mandibular canal occasionally present an intimate anatomical relationship with the inferior alveolar nerve (IAN), and so the nerve may be damaged during the extraction procedure (2). The rationale for considering coronectomy is based on the assessed risk of IAN injury related to impacted third molar surgery (3).

In 1989, Knutsson *et al*. (4) described coronectomy as a new alternative for removing impacted third molars in close proximity to the IAN. The coronectomy technique consists of removing the crown of the tooth while maintaining the retained vital roots that are intimately related with the IAN (5). In recent years, this technique has been indicated for cases characterized by close contact between the mandibular third molar and the IAN, where complete removal could cause injury to the nerve (6).

Coronectomy seems to be safer than conventional extraction in terms of the incidence of nerve damage, and comparable in terms of the incidence of dry socket and postoperative infection (7). According to a systematic review (8), the coronectomy success rate is 93%, and success depends on both the patient characteristics and the technique used. In turn, the incidence of injury to the IAN is reportedly lower in failed coronectomies than in conventional surgical extractions.

Coronectomy has been associated to minimal morbidity in terms of pain, infection or the development of disease conditions (9). Long-term postoperative evaluation is necessary to assess the probability of root migration and the rates of extraction or infection (10). Recent studies and meta-analyses of coronectomies (6) described the technique as a good option for preventing nerve damage, and it does not seem to be related to a higher incidence of postoperative infections compared to conventional surgical extraction of mandibular third molars. Despite this, coronectomy is not accepted by all professionals, and is still the subject of much debate. More long-term studies are thus needed.

The aim of the present retrospective case series study was to evaluate the intraoperative and short- and long-term complications, and the clinical and radiological outcomes of impacted mandibular third molars in intimate contact with the IAN and treated with the coronectomy technique.

## Material and Methods

- Study design

A retrospective clinical case series study was made to analyze the clinical complications and radiological outcomes of the coronectomy technique in treating impacted mandibular third molars indicated for extraction. Approval was obtained from the Clinical Research Ethics Committee of the University of Valencia (Valencia, Spain) (Ref. UV-INV_ETICA-1617553). Each patient provided written informed consent to authorize the use of their medical history and radiographic tests for educational or research purposes.

- Decision making before the surgical procedure

The patients with an indication of mandibular third molar surgical extraction underwent anamnesis, a clinical oral exploration and a preliminary panoramic radiographic evaluation. The following radiographic signs that are significantly associated with an increased risk of IAN injury in mandibular third molar extractions were evaluated (11): deviation or narrowing of the mandibular canal; periapical radiolucency; narrowing, darkening and curving of the tooth roots; and loss of lamina dura over the wall of the mandibular canal. If any of the previous signs were present, a cone-beam computed tomography (CBCT) study was made for planning the intervention (12).

Mandibular third molars with a high risk of IAN injury based on the panoramic radiographs and CBCT findings were selected for coronectomy. Coronectomy was not performed in the following situations: third molar with caries that compromised the pulp-dentin complex, a non-vital third molar, cystic lesions related to the mandibular third molar, local active infection and systemic disorders contraindicating surgery.

All patients were informed of the possible complications that might occur if complete extraction of the third molar were performed, or if the roots were left in place. In the case of coronectomy, the patients were made fully aware of the potential risks of postoperative complications (infection, IAN injury, severe pain, bleeding and swelling), and of the additional possibility of second surgery to remove the roots, either early or late after the operation (13).

A search was made of the database of the Oral Surgery Unit (Department of Stomatology, University of Valencia), covering the period between 2011 and 2020. Approximately 2000 impacted mandibular third molar extractions and 39 coronectomies were performed during this period.

The inclusion criteria for mandibular third molar coronectomy were the availability of a complete set of radiographic tests (a preoperative, immediate postoperative and at least a two-year panoramic radiographic study, and a preoperative CBCT scan) and a complete clinical history including patient age, sex, systemic diseases, allergies and medications, intraoperative complications (mobilization of the root fragments and damage to adjacent structures), short-term postoperative complications (injury of the IAN or lingual nerve based on the detection of sensory alterations in the respective nerve territories, and postoperative infection [pus drainage]), and long-term postoperative complications (need to extract the remnant roots, infection or oral exposure). The exclusion criteria in turn were a lack of follow-up or incomplete clinical and radiographic data.

- Surgical technique

A preoperative planning was established based on the preoperative panoramic radiographic and CBCT study to evaluate the exact relationship between the mandibular canal and the mandibular third molar roots. All surgical procedures were performed by the same operators (MPD or DPO) according to the standard protocol for coronectomies (5,14).

The coronectomies were carried out under local anesthesia (4% articaine with 1:100,000 adrenaline). A triangular incision was made with buccal-distal discharge in the retromolar zone and with buccal-mesial discharge in the second molar. A mucoperiosteal flap was raised and retained with a Minnesota retractor, which causes minimal damage to the mucosa and helps secure correct angulation of the cutter. The necessary bone was removed to obtain a correct visual field. The tooth was exposed to the level of the cementoenamel junction. Coronectomy was performed with a straight handpiece and round bur at 1 mm below the cementoenamel junction, separating the crown from the intact and retained roots. Perpendicular and complete crown removal of the tooth without perforating the lingual cortical bone was ensured. Once the crown was removed, the root surface was reduced 2-4 mm below the bone margins using a round bur, eliminating all enamel and dentin tips (8,15). A minimum quantity of root (2 mm to the IAN) was left. Root canal treatment was not performed in any case. Next, a dental curette was used to remove any soft follicular tissue in the surgical bone defect. The socket was irrigated with saline solution and closed with 4-0 polyamide simple sutures (Supramid, Proclinic, Spain). Primary and tension-free closure was desirable whenever possible.

**Figure 1:**
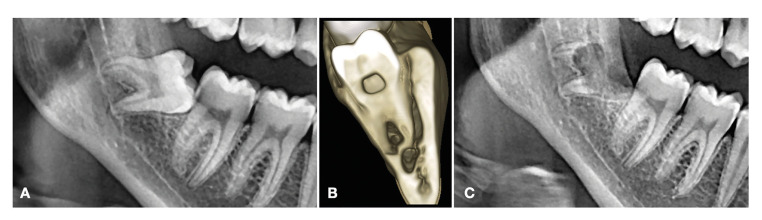
A) Right lower third molar appearing to be intimately related to the inferior alveolar nerve in the preoperative panoramic radiographic study. B) The inferior alveolar nerve crossed the two mesial roots of the right lower third molar. C) Postoperative panoramic radiographic view following coronectomy.

In all cases, a postoperative panoramic radiograph was obtained to visualize the root fragments after the coronectomy procedure (Fig. 1). The drug treatment of choice was amoxicillin 500 mg (3 Tablets a day for 7 days) as antibiotic medication and ibuprofen 600 mg (one Tablet every 8 hours for 3 days) as antiinflammatory and analgesic medication. A soft and cold diet for 24 hours was prescribed, with local cold in the form of an ice pack applied to the cheek in the surgical area for about 15-20 minutes during the first hour after surgery. Oral hygiene measures were adopted (0.12% chlorhexidine mouth rinse 3 times daily until removal of the sutures one week after surgery).

- Follow-up

**Figure 2:**
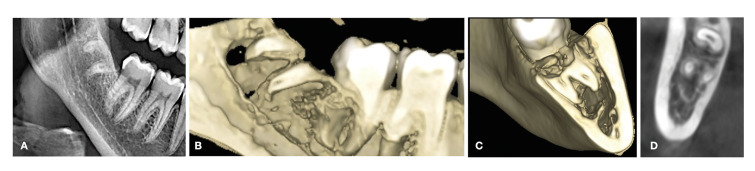
A) Panoramic radiographic study after 24 months of follow-up. The migration of the roots can be noted. B) CBCT reconstruction after 24 months of follow-up, showing the separation between the inferior alveolar nerve and the mesial root. C) CBCT reconstruction after 24 months of follow-up, showing the separation between the inferior alveolar nerve and the mesial root. D) Parasagittal view showing the separation between the apex and the inferior alveolar nerve.

All patients were controlled one week and one month after surgery. An annual panoramic radiograph and general and oral exploration were subsequently performed for each year (Fig. 2).

- Clinical and radiological evaluation

The clinical outcomes were retrieved from the medical records of each patient. The clinical complications were divided into three groups: intraoperative complications (mobilization of the root fragments and damage to adjacent structures), short-term postoperative complications (sensory alterations of the IAN or lingual nerve, and postoperative infection during the first month), and long-term postoperative complications (oral exposure of the root or infection after the first month). The need to extract the remnant roots due to infection or oral exposure was also recorded.

The radiological outcomes were measured from the different panoramic radiographs and the CBCT scan. The following radiological information was collected: the position of the IAN with respect to the roots (inter-radicular, buccal, lingual, apical), root shape, eruption status, third molar position and direction of migration of the radicular complex (no migration, mesial, distal, occlusal).

## Results

Approximately 2000 mandibular third molar removals were identified in the database, of which 43 molars in 38 patients were extracted via coronectomy. Four third molars in four patients were excluded due to a lack of follow-up. Finally, 39 third molars in 34 patients (15 men and 19 women) were included in the study. The mean age was 36 years (range 22-77), and the mean duration of follow-up was 28 months (range 12-84). The patient characteristics are shown in Table 1.

**Table 1:**
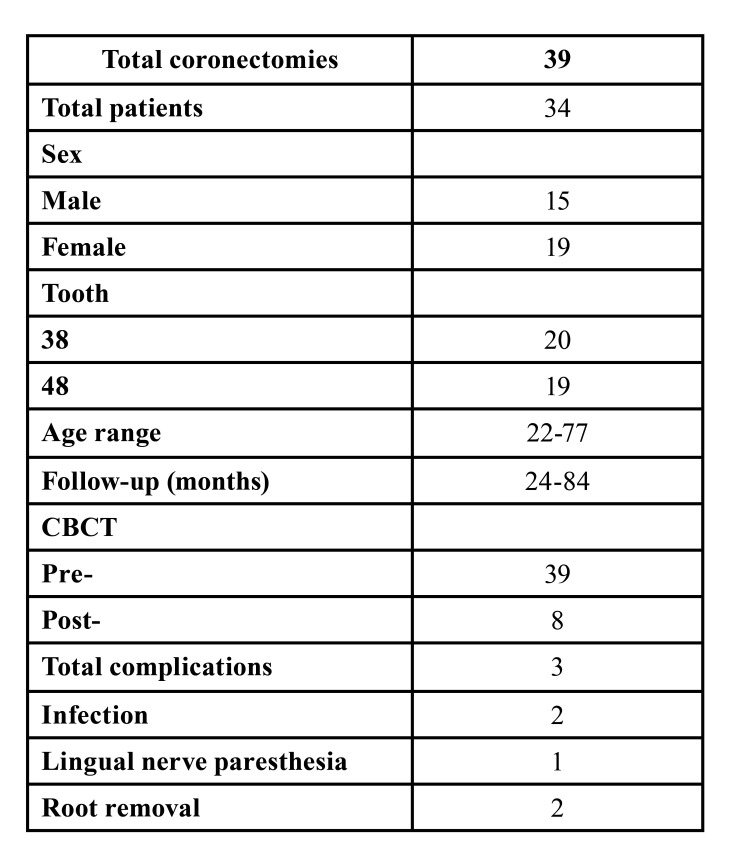
Patient characteristics.

- Clinical outcomes

Most of the patients showed good healing after treatment, though three of them experienced short-term complications. Two patients developed infection associated with intense pain, swelling and trismus during the postoperative period. One patient worsened at two weeks post-extraction and improved significantly after re-intervention to remove the root fragment with one week of systemic antibiotic treatment (amoxicillin 875 mg and clavulanic acid 125 mg, one Tablet every 8 hours for 7 days). Removal proved easier than first surgery because the root fragment presented migration and mobility. The other patient suffered severe infection due to a pterygomandibular abscess and required hospital admission and intensive care (Valencia University Clinic Hospital, Valencia, Spain); intraoral drainage and root extraction were carried out, and the complication was resolved. Lastly, one patient reported a tingling sensation due to paresthesia of the lingual nerve; at examination, right lingual hypoesthesia was noted, possibly due to lingual retraction. The hypoesthesia subsided within three weeks. There were no cases of permanent injury of the IAN, nor any long-term complications.

- Radiological evaluation

A panoramic radiographic study was made 24 months after surgery, and only 8 patients also underwent a postoperative CBCT scan. Root migration was detected in 11 cases (8 in the mesial direction and 3 in the occlusal direction), while 28 cases showed no migration. The extraction of roots exposed to the oral cavity secondary to migration was necessary in two cases. The radiological characteristics are described in Table 2.

**Table 2:**
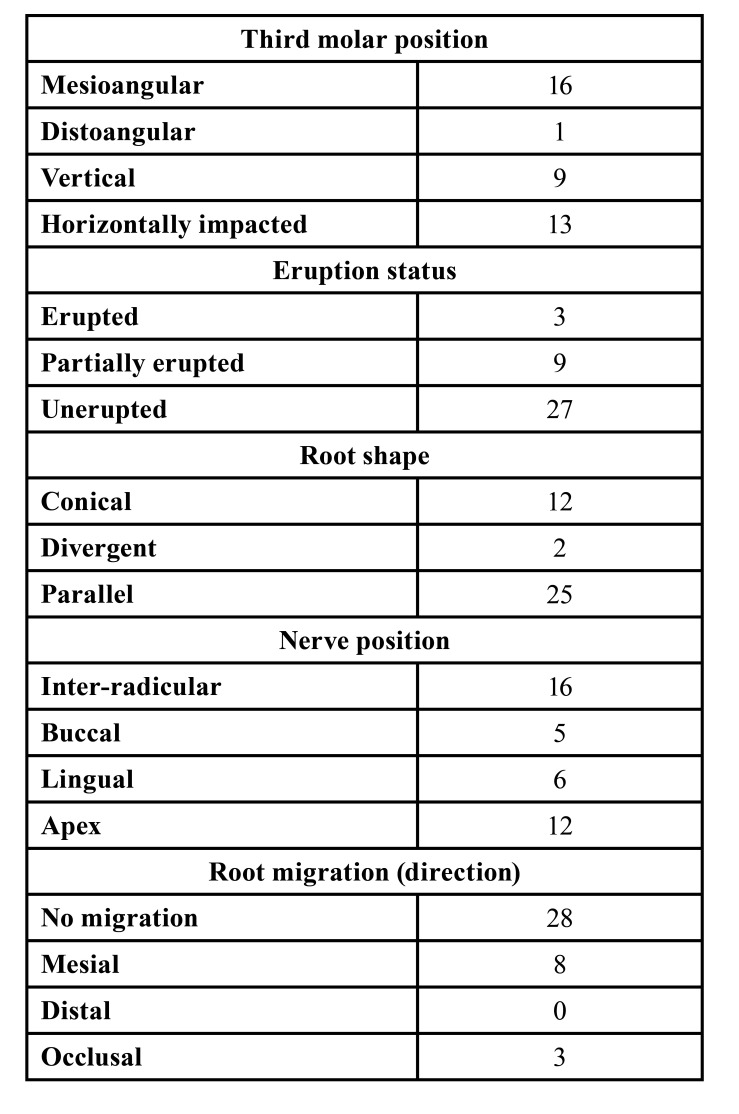
Radiological characteristics.

## Discussion

The main objective of this clinical and radiological retrospective case series study was to evaluate the intraoperative, short-term and long-term complications and the clinical and radiological outcomes of the coronectomy technique applied to impacted mandibular third molars in intimate contact with the IAN.

The coronectomy procedure has been reported as an effective alternative treatment for avoiding IAN injury in high-risk patients subjected to surgical extraction of mandibular third molars (16). The coronectomies in this study were performed in cases where the IAN presented some radiographic features indicative of a high risk of nerve injury according to Rood and Shehab (12).

Regarding the surgical procedure, some technical variations have been described in the scientific literature, such as the use of different cutting instruments (17), the possibility of concurrent root canal treatment (18), or the application of guided bone regeneration techniques (19).

Different instruments can be to used for performing the coronectomy. According to an *in vitro* study (17), the round bur with a conventional straight handpiece used in the present study is one of the best options in terms of heat generation, preparation time, coronectomy success and cutting surface quality, compared to the surgical handpiece high-speed rotating and piezoelectric instruments. The quantity of remnant root fragment could be another factor to be taken into account. In the present study, coronectomy was performed to approximately 2 mm from the IAN in order to avoid nerve damage. In the current scientific literature, coronectomy is circumscribed to the tooth crown (5,7).

The efficacy of concurrent root canal treatment of the retained roots has been the subject of debate. The only randomized clinical trial to date (20) has demonstrated that concurrent root canal treatment resulted in a higher postoperative infection rate (7 of 8 cases; 87.5%) than when not performing root canal treatment (1 of 8 cases; 12.5%). A histological study (21) in turn analyzed 26 roots derived from anterior coronectomies that were removed after migration and oral exposure with inflammation of the surrounding soft tissue. The results showed that all the coronectomies still contained vital pulp tissue, with no evidence of periradicular inflammation. The addition of mineral trioxide aggregate (MTA) above the pulp chamber has been described in a case report to demonstrate the possible effectiveness of this method for tertiary dentin formation between the pulp and the MTA under the cement (22). In the present study, pulp treatment was not performed, and a low incidence of complications was recorded.

Guided bone regeneration techniques have been also implemented in a split-mouth randomized clinical trial comprising a sample size of 48 bilateral coronectomies with a follow-up of two years (19). Root migration was significantly less in the guided bone regeneration group (11.3 mm) than in the control group (3.51.9 mm). In the present study, no guided bone regeneration was used, and migration of the roots occurred in 11 of the 39 cases. The infection rate was 2.2% and 4.3% with and without guided bone regeneration, respectively - the difference failing to reach statistical significance.

The coronectomy success rate depends on the survival of the retained root fragments, with successful formation of bone over the roots and no presence of symptoms (14). According to a systematic review (8), 125 of 2087 included coronectomies failed, yielding a failure rate of 7%. The detected causes of failure in the systematic review comprised pulpitis (3%), enamel retention (7%), wound dehiscence (9%), infection (8%), migration/exposure of root (33%) and root mobility (36%). The success rate in the present study is consistent with the reviewed literature (8,23,24). However, the detected cause of coronectomy failure was postoperative infection, requiring re-intervention to extract the remaining roots in two cases. The role of coronectomy in relation to infection is difficult to establish. It is possible that the remnant root fragment facilitated infection, though infection can also occur after the extraction of an impacted mandibular molar.

The complications can be divided into three categories: intraoperative (mobilized fragments of root or damage to adjacent structures), short-term postoperative (alveolar osteitis, pain, infection or nerve injury) and long-term postoperative (migration and oral eruption of roots) (23). No intraoperative, three short-term postoperative (7.6%) and some long-term postoperative complications were detected (2 cases required extraction due to oral exposure and 9 due to tooth migration). The long-term complications were mild, because migration of the roots is an asymptomatic process, and oral exposure occurred without infection. The global incidence of complications was low. Previous studies on the total extraction of impacted mandibular third molars in our Department have evidenced low infection rates (2-4%) (24,25). In the present study, two cases (5.18%) showed infection. Oral exposure was detected in between 0-1.6% of all cases according to a systematic review (26), though in the present study the incidence was 5.12%.

According to two systematic reviews (26,27), the coronectomy procedure of seems to be safer than total extraction in terms of a lesser risk of IAN damage during the surgical treatment of impacted mandibular third molars. Some factors have been identified as influencing variables in the incidence of nerve injury: the anatomical position of the tooth requiring extraction, the age of the patient, nerve exposure, the access technique used for extraction, and surgeon experience (28). In the present study, only one case (2.56%) presented temporary paresthesia. The IAN was not affected. In a systematic review (8), a greater overall incidence of IAN injury was detected in failed procedures (2.6%) than in successful operations (0.5%). In the present case series, the two coronectomies that failed due to postoperative infection did not show nerve damage. The infective complications rate was 5.12% (2 infections out of 39 coronectomies). In a recent systematic review (29) comprising 5613 third molar extractions, the infection rate was 4.3% when antibiotics were prescribed and 7.5% when they were not. Thus, the infection rate after coronectomy in this study is similar to that observed after the conventional surgical extraction of included mandibular third molars.

Regarding migration of the remnant roots, 28 roots remained intact and 11 showed migrations. Kohara *et al*. (30) concluded that the roots migrate in the first two years, and rarely after that. In a systematic review (6), the mean distance of root migration was 2 mm over two years.

The limitations of the present study were the small sample size involved, the lack of a control group, and the possibility of underestimating the complications due to retrospective analysis of the sample. Randomized clinical trials comparing conventional extraction of included third molars versus coronectomy are necessary in order to improve the evidence in this field.

## Conclusions

In the present case series of 39 impacted mandibular molars in intimate relation to the IAN and subjected to coronectomy, the number of complications was low (2 infections and one case of temporary lingual paresthesia), and no permanent sensory alterations were caused. Prospective studies, especially randomized clinical trials, are needed to compare this technique versus conventional extraction.
